# CD8 T Cells Show Protection against Highly Pathogenic Simian Immunodeficiency Virus (SIV) after Vaccination with SIV Gene-Expressing BCG Prime and Vaccinia Virus/Sendai Virus Vector Boosts

**DOI:** 10.1128/JVI.01718-20

**Published:** 2021-01-28

**Authors:** Seiichi Kato, Hisatoshi Shida, Tomotaka Okamura, Xianfeng Zhang, Tomoyuki Miura, Tetsu Mukai, Makoto Inoue, Tsugumine Shu, Taeko K. Naruse, Akinori Kimura, Yasuhiro Yasutomi, Kazuhiro Matsuo

**Affiliations:** a Tsukuba Primate Research Center, National Institutes of Biomedical Innovation, Health and Nutrition, Ibaraki, Japan; b Research & Development Department, Japan BCG Laboratory, Tokyo, Japan; c Institute for Genetic Medicine, Hokkaido University, Sapporo, Japan; d Institute for Frontier Life and Medical Sciences, Kyoto University, Kyoto, Japan; e Leprosy Research Center, National Institute of Infectious Diseases, Tokyo, Japan; f ID Pharma Co., Ltd., Ibaraki, Japan; g Tokyo Medical and Dental University, Tokyo, Japan; h Department of Immunogenetics, Institute of Tropical Medicine, Nagasaki University, Nagasaki, Japan; Emory University

**Keywords:** HIV, SIV, recombinant BCG, recombinant vaccinia, CTL, human immunodeficiency virus, simian immunodeficiency virus

## Abstract

Toward development of a dual vaccine for human immunodeficiency virus type 1 (HIV-1) and tuberculosis infections, we developed a urease-deficient bacillus Calmette-Guérin (BCG) strain Tokyo172 (BCGΔurease) to enhance its immunogenicity. BCGΔurease expressing a simian immunodeficiency virus (SIV) Gag induced BCG antigen-specific CD4^+^ and CD8^+^ T cells more efficiently and more Gag-specific CD8^+^ T cells. We evaluated its protective efficacy against SIV infection in cynomolgus monkeys of Asian origin, shown to be as susceptible to infection with SIVmac251 as Indian rhesus macaques. Priming with recombinant BCG (rBCG) expressing SIV genes was followed by a boost with SIV gene-expressing LC16m8Δ vaccinia virus and a second boost with SIV Env-expressing Sendai virus. Eight weeks after the second boost, monkeys were repeatedly challenged with a low dose of SIVmac251 intrarectally. Two animals out of 6 vaccinees were protected, whereas all 7 control animals were infected without any early viral controls. In one vaccinated animal, which had the most potent CD8^+^ T cells in an *in vitro* suppression activity (ISA) assay of SIVmac239 replication, plasma viremia was undetectable throughout the follow-up period. Protection was confirmed by the lack of anamnestic antibody responses and detectable cell-associated provirus in various organs. Another monkey with a high ISA acquired a small amount of SIV, but it later became suppressed below the detection limit. Moreover, the ISA score correlated with SIV acquisition. On the other hand, any parameter relating anti-Env antibody was not correlated with the protection.

**IMPORTANCE** Because both AIDS and tuberculosis are serious health threats in middle/low-income countries, development of a dual vaccine against them would be highly beneficial. To approach the goal, here we first assessed a urease-deficient bacillus Calmette-Guérin (BCG) for improvement of immunogenicity against both Mycobacterium tuberculosis and SIV. Second, we demonstrated the usefulness of Asian-origin cynomolgus monkeys for development of a preclinical AIDS vaccine by direct comparison with Indian rhesus macaques as the only validated hosts that identically mirror the outcomes of clinical trials, since the availability of Indian rhesus macaques is limited in countries other than the United States. Finally, we report the protective effect of a vaccination regimen comprising BCG, the highly attenuated vaccinia virus LC16m8Δ strain, and nontransmissible Sendai virus as safe vectors expressing SIV genes using repeated mucosal challenge with highly pathogenic SIVmac251. Identification of CD8^+^ T cells as a protective immunity suggests a future direction of AIDS vaccine development.

## Abstract (precis)

Because both AIDS and tuberculosis are serious health threats in middle/low-income countries, development of a dual vaccine against them would be highly beneficial. To approach the goal, here we first assessed a urease-deficient bacillus Calmette-Guérin (BCG) for improvement of immunogenicity against both Mycobacterium tuberculosis and SIV. Second, we demonstrated the usefulness of Asian-origin cynomolgus monkeys for development of a preclinical AIDS vaccine by direct comparison with Indian rhesus macaques as the only validated hosts that identically mirror the outcomes of clinical trials, since the availability of Indian rhesus macaques is limited in countries other than the United States. Finally, we report the protective effect of a vaccination regimen comprising BCG, the highly attenuated vaccinia virus LC16m8Δ strain, and nontransmissible Sendai virus as safe vectors expressing SIV genes using repeated mucosal challenge with highly pathogenic SIVmac251. Identification of CD8^+^ T cells as a protective immunity suggests a future direction of AIDS vaccine development.

## INTRODUCTION

The human immunodeficiency virus type 1 (HIV-1)/AIDS epidemic continues to spread worldwide (1). Although preexposure prophylaxis (PrEP), for which uninfected individuals take a daily antiretroviral pill to reduce their risk of becoming infected, has been trialed, its efficacy has not been conclusively demonstrated and probably depends on strict adherence (2). Antiretroviral therapy (ART) has been reported to be effective in prevention of HIV-1 infection (3), but people unaware that they are infected are a major source of infection. Thus, the development of an effective vaccine for use in conjunction with ART is considered to be the most promising way to end the HIV epidemic.

Although effective vaccines and their regimens have yet to be developed, recent studies have revealed promising immune responses that prevent HIV-1 infection. Analyses of the RV144 vaccine trial showed that the heterologous prime-boost regimen reduced HIV-1 acquisition by 31% (4) and identified the induction of antibody with antibody-dependent cell-mediated cytotoxicity (ADCC) activity against the V2 region of gp120 as a correlate of protective immunity (5). Moreover, isolation of broadly neutralizing monoclonal antibody (bNAb) clones from HIV-1-infected individuals revealed the existence of common neutralizing epitopes across different clades of HIV-1 (6–8), and the protection of macaques against simian-human immunodeficiency virus (SHIV) challenge by passive immunization with relatively low concentrations of bNAbs suggests that the development of broadly effective vaccines is achievable (9). In addition, effector memory T cells (Tem cells) detectable for long periods after vaccination have also been shown to be effective at restricting infection by antibody-resistant simian immunodeficiency virus (SIV) at the virus entry site. This was achieved by vaccine approaches that result in persistent expression of antigens in vaccinated macaques, such as the use of attenuated SIVs (10) or a SIV gene-expressing cytomegalovirus (CMV) vector, which result in continuous immunological stimulation (11, 12). However, getting strategies of this kind into clinical trials will require significant hurdles to be overcome, not least because their use in humans is unprecedented.

HIV-1 infection is most prevalent in middle/low-income countries where other infectious diseases, including tuberculosis, are also a serious health threat; thus, the development of a dual vaccine against HIV-1 and Mycobacterium tuberculosis would be highly beneficial. Although bacillus Calmette-Guérin (BCG) vaccination in infancy reduces infection of M. tuberculosis and progression to active disease before adolescence, such vaccination is not effective for pulmonary tuberculosis in adults. Therefore, booster vaccination in adolescence has been under consideration. Several novel vaccines against M. tuberculosis have been developed, including ones for which new technologies were applied; however, as a booster vaccine, BCG remains the most effective in clinical trials (13), and new vaccination route/administration methods, such as pulmonary mucosal delivery and intravenous administration, have been shown to enhance the effect of BCG in preventing pulmonary tuberculosis (14, 15).

To develop HIV-1 vaccine, we have adopted an approach including modifications of two effective, safe, economical, existing platforms that include BCG. In particular, Tokyo172 strain is less reactogenic than other BCG strains (16). BCG persists for long periods after vaccination, suggesting that it can elicit long-lasting immunity. However, previous studies showed that vaccination with BCG-expressing SIV genes was not potent enough to prevent infection on challenge with SIV (17). Thus, means of improving antigenicity have been investigated, for example, by deleting urease from BCG. Urease is involved in the neutralization of the phagosome in which BCG is harbored. Its depletion allows for rapid phagosome acidification and promotes phagolysosome fusion. As a result, the urease-deficient BCG (BCGΔurease) promotes release of its antigens into the cytosol, leading to efficient trapping by the proteasome for presentation to induce a cytotoxic T-lymphocyte (CTL) response (18).

As another platform, we engaged vaccinia virus (VV) strain LC16m8Δ (19, 20), which is an improved strain in preventing spontaneous generation of virulent revertants from strain LC16m8, the Japanese smallpox vaccine, which has been administered to over 100,000 people without any serious adverse effect (21–23). We previously demonstrated in mice that a vaccination regimen of priming with recombinant BCGs (rBCGs) expressing the SIV *gag* gene, followed by boosting with the replication-competent VV strain LC16m8Δ, efficiently elicited anti-Gag CD8^+^ T cells that were maintained as Tem cells for a long period (24, 25). Therefore, a regimen including rBCG and LC16m8Δ recombinants may be applicable to humans as a dual vaccine for tuberculosis and HIV-1.

Additionally, we used an envelope-expressing Sendai virus vector (SeV-Env) (26) as a booster vaccine to maximize elicitation of anti-envelope antibody. We previously showed in mice that priming with LC16m8Δ-Env followed by an SeV-Env boost effectively elicited antibodies against the HIV-1 gp160 Env protein (27, 28). For safety, the SeV vector was a nontransmissible and replication-competent version; however, a small clinical trial showed that the transmissible and replication-competent vector did not cause serious complications (29).

As a precursor to HIV-1 vaccines, efficacy of the immunization regimen can be tested in primates using SIV, and it is critically important to use antibody-resistant (tier 2) SIV strains, such as SIVmac239 or SIVmac251, as a challenge virus, because HIV vaccine cognate immunogens based on the protective effects seen with the antibody-sensitive SIVsmE660 (30) and SHIV89.6 (31) strains were not effective in the human HVTN505 (http://www.niaid.nih.gov/news/newsreleases/2013/Pages/HVTN505April2013.aspx) and STEP trials (32). In particular, repeated mucosal challenge with SIVmac251 represents a stringent preclinical model of a highly pathogenic, neutralization-resistant virus swarm and mimics sexually transmitted HIV-1 in humans (33, 34). Moreover, Indian rhesus macaques with defined major histocompatibility complex (MHC) genotypes most sensitive to SIV infection are the only validated hosts that identically mirror the outcomes of clinical trials such as RV144 and HVTN702 (4, 33, 34). However, the availability of Indian rhesus macaques is limited in countries other than the United States, greatly hampering HIV-1 vaccine development. Identifying other appropriate monkey species would promote the progress of HIV-1 vaccine studies. Cynomolgus monkeys of Asian origin might be an alternative model animal, as they are highly susceptible to SIVmac239 and SIVmac251 (35), although those from Mauritius are less susceptible to SIVmac251 (36). A direct comparison between Asian cynomolgus monkeys and Indian rhesus macaques as models for HIV vaccine development, however, is lacking.

In this study, we first assessed whether the immunogenicity and pathogenicity of BCG were improved by mutation in the urease gene. Second, the susceptibility of Asian cynomolgus monkeys to SIVmac251 was compared with that of Indian rhesus macaques. Finally, the efficacy of a vaccination regimen comprising priming with rBCGΔurease expressing SIV genes (rBCGΔurease-SIV), boosting with VV strain LC16m8Δ recombinants (m8Δ-SIV), and, to maximize elicitation of anti-Env antibody, further boosting with SeV-Env was analyzed after repeated mucosal challenge with pathogenic SIVmac251.

## RESULTS

### Comparison of pathogenicities and immunogenicities of various BCGs.

**FIG 1:**
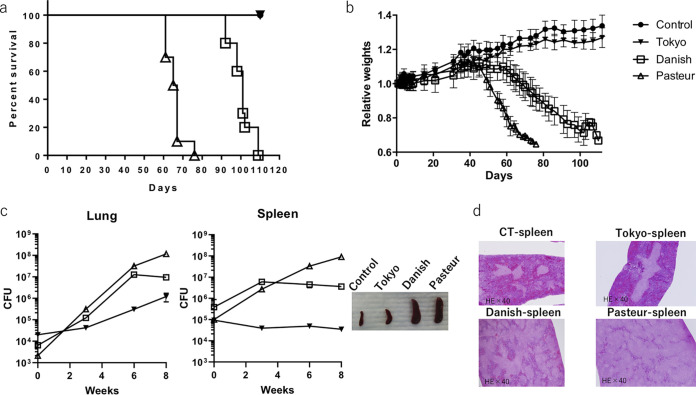
Pathogenicity of various BCG strains in SCID mice. SCID mice were inoculated intravenously with 10^7^ CFU of each BCG, which was calculated by a calibration curve between the OD of 7H9-ADC liquid culture of the BCG strains and the numbers of colonies on a Middlebrook 7H10 agar plate containing oleic albumin dextrose catalase supplement (Becton, Dickinson and Co., Tokyo, Japan). (a) Survival of SCID mice uninfected (●) or infected with the Danish (□), Pasteur (△), or Tokyo172 (▾) strain of BCG (*n* = 10 for each). (b) Body weights of SCID mice over time after BCG infection. (c) The numbers of living BCG that persisted in the lungs and spleens of SCID mice. Photographs of spleens at 8 weeks after infection show splenomegaly of Danish and Pasteur strain-infected SCID mice. (d) Hematoxylin-and-eosin (HE)-stained paraffin sections of mouse spleens prepared at 8 weeks after infection.

First, we compared the pathogenicities of various BCG strains (Danish, Pasteur, and Tokyo172) used as vaccines against tuberculosis worldwide but for which all have had occasional adverse events reported. A paper reported that the number of CFU of BCG Japanese (Tokyo) was much lower than that of BCG Pasteur in both BALB/c and C3H mouse spleens at 2 to 12 weeks postinoculation (37). This indicates that BCG Tokyo is cleared faster and is less toxic than BCG Pasteur in such normal mice.To compare and assess the pathogenicities of BCG strains precisely to select the most appropriate one as an HIV vaccine vector, we tested their toxicities in severe combined immunodeficiency (SCID) mice.BCG (10^7^ CFU) was inoculated intravenously into SCID mice, and survival of the mice was monitored. All mice inoculated with the Danish and Pasteur strains ultimately died, while Tokyo172-infected mice survived and with increased body weight (Fig. 1a and b). In general, mycobacterial multiplicity and persistence *in vivo* have been analyzed by measurement of CFU in lung and spleen in relation to local and systemic immunity, respectively. The number of bacteria increased over time in the lungs of all mice, but this was true in the spleens of mice infected with the Danish and Pasteur strains but not in the spleens of Tokyo172-infected mice. Splenomegaly was apparent in mice infected with the Danish and Pasteur strains but not in those infected with Tokyo172 (Fig. 1c). Cross-sections of spleen stained with hematoxylin and eosin revealed little damage with Tokyo172 infection, in contrast to the severe damage seen with Danish and Pasteur BCG infection (Fig. 1d). These results are consistent with fewer adverse events being reported in Tokyo172-vaccinated persons than in those vaccinated with the Danish or Pasteur strain (16).

**FIG 2:**
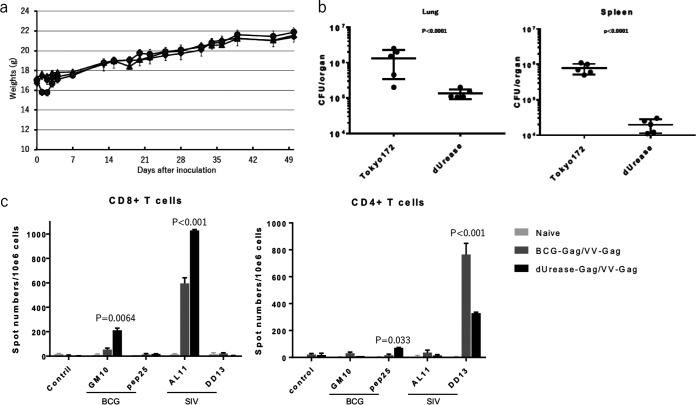
Pathogenicity and immunogenicity of urease-deficient BCG Tokyo172 (BCGΔurease) harboring plasmid bearing the SIV *gag* gene. (a) Body weights of SCID mice over time after wild-type BCG (▴), BCGΔurease (●), or mock (◆) infection (*n* = 5 for each). SCID mice were inoculated intravenously with 10^7^ CFU of each BCG. One of the tubes containing frozen BCG was molten, and the number of CFU was measured by the colony assays. (b) Numbers of living BCG that persisted in the spleens and lungs of SCID mice at 8 weeks after infection. (c) ELISPOT assay of splenocytes prepared from C57BL/6J primed with BCG-SIV-Gag and boosted with m8Δ-SIV-Gag.

Next, we examined the pathogenicity of the BCGΔurease version of the Tokyo172 strain (38). The body weight of the BCGΔurease-infected SCID mice increased over time at the same rate as that of wild-type Tokyo172-infected mice (Fig. 2a), while there was less BCGΔurease than wild-type Tokyo172 persistent in the lung and spleen (Fig. 2b). To assess the immunogenicity of BCGΔurease and its capacity to induce immunity to foreign antigens, we primed C57BL/6J mice with BCGΔurease containing a plasmid bearing the SIV *gag* gene and boosted with LC16m8Δ expressing the *gag* gene. An enzyme-linked immunospot (ELISPOT) assay revealed that BCGΔurease elicited more gamma interferon (IFN-γ)-producing CD4^+^ and CD8^+^ T cells specific to BCG antigens. Moreover, BCGΔurease elicited more SIV Gag-specific CD8^+^ T cells but fewer CD4^+^ T cells than wild-type BCG, which are characteristics suitable for an HIV-1/SIV vaccine (Fig. 2c).

### Susceptibility of cynomolgus monkeys to SIVmac251.

To directly compare the susceptibilities of cynomolgus monkeys of Asian origin with Indian rhesus macaques, we repeatedly inoculated a low dose (300 50% tissue culture infective doses [TCID_50_]) of SIVmac251, a highly virulent and pathogenic SIV strain, intrarectally and quantified the viral load by quantitative PCR. As shown in Fig. S1 in the supplemental material, all seven cynomolgus monkeys acquired SIV after four inoculations, while one of three rhesus macaques required a further three inoculations to be infected. Moreover, peak viral loads in cynomolgus monkeys were higher than those in Indian rhesus macaques, while viral loads at set point in both groups were not significantly different. Even in a comparison with results of a previous report (36), 3.6 × 10^7^ copy numbers/ml of mean plasma viral load at peak in the cynomolgus monkeys was comparable to ∼2 × 10^7^ copy numbers/ml in Indian rhesus macaques. The mean plasma viral load at set point in the cynomolgus monkeys of 1.8 × 10^5^ copy numbers/ml is similar to ∼5 × 10^5^ copy numbers/ml in Indian rhesus macaques. Thus, these results suggest that the Asian cynomolgus monkeys are as susceptible to SIVmac251 as Indian rhesus macaques, although a study with more animals is necessary to confirm this.

### Vaccination and low-dose challenge with SIVmac251.

**FIG 3:**
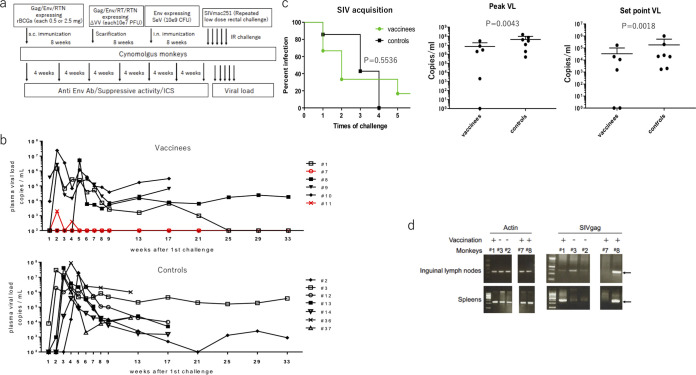
Vaccination of cynomolgus monkeys and challenge with SIVmac251. (a) Schematic vaccination and challenge protocols. Arrows represent time of vaccination or challenges. s.c., subcutaneous; i.n., intranasal; IR, intrarectal. (b) Outcome of repeated, limiting-dose intrarectal SIVmac251 challenge. Upper and lower panels show plasma viral loads of vaccinated and control monkeys, respectively. (c) The rates of SIVmac251 acquisition per exposure were compared using the log rank test of the discrete-time proportional hazards model, and viral loads (VL) at peak and set point (17 weeks after the first challenge, except 13 weeks after the first challenge for two control monkeys) were compared using the Mann-Whitney test. (d) Proviral DNA loads in lymphoid tissues prepared at euthanasia corresponding to the last time point of viral load measurements were detected by PCR. Information for the monkeys used in this experiment, including sex, age, and MHC typing data, is given in Table 3.

Three cynomolgus monkeys (monkeys 1, 7, and 8) were initially vaccinated subcutaneously with a mixture of 0.5 mg of each rBCGΔurease expressing Gag, a fusion protein of Rev, Tat, and Nef (RTN), or gp120, and three monkeys (9, 10, and 11) were initially vaccinated with a mixture of 2.5 mg of each rBCGΔurease. The monkeys were then boosted by skin scarification with a mixture containing 10^7^ PFU of VV m8Δ expressing Gag, RTN, gp160, or Pol with an 8-week interval. Finally, they were further boosted with 10^9^ CFU of SeV-Env with an 8-week interval (Fig. 3a). Eight weeks after the final vaccination, vaccinated and control cynomolgus monkeys were challenged intrarectally with weekly low doses (300 TCID_50_) of SIVmac251 to assess the protective efficacy of the vaccine regimen. Plasma samples were assayed for SIV RNA by quantitative PCR 7 days after each challenge. SIV was administered weekly 5 times. All seven control animals became infected after four challenges as described above and maintained a constant viral load (Fig. 3b). Rates of viral acquisition were not significantly different between controls and vaccines, but mean viral loads at both the peak and the set point were significantly lower in vaccinated monkeys than in controls (Fig. 3c). One of the six vaccinated monkeys (monkey 7) did not acquire infection with SIV even after five challenges, and virus was never detected during the whole observation period up to 75 weeks after the first challenge. Another (monkey 11) acquired a low titer of SIV after two challenges but subsequently suppressed the viral load below the detection limit. The viral load of monkey 1, which acquired SIV after two challenges, was reduced to below the detection limit 25 weeks after challenge and was maintained at an undetectable level thereafter (Fig. 3b).

At the end of these experiments, the animals were sacrificed, and viral genomes from various tissues were examined by nested PCR (Fig. 3d). Viral genomes were undetectable in any examined tissues of monkey 7, suggesting sterile protection. In contrast, all other animals, including monkey 11, which acquired but suppressed SIV below the detection limit, contained SIV genomes in secondary lymphoid organs (data not shown).

### SIV-specific antibody elicited by vaccination.

**FIG 4:**
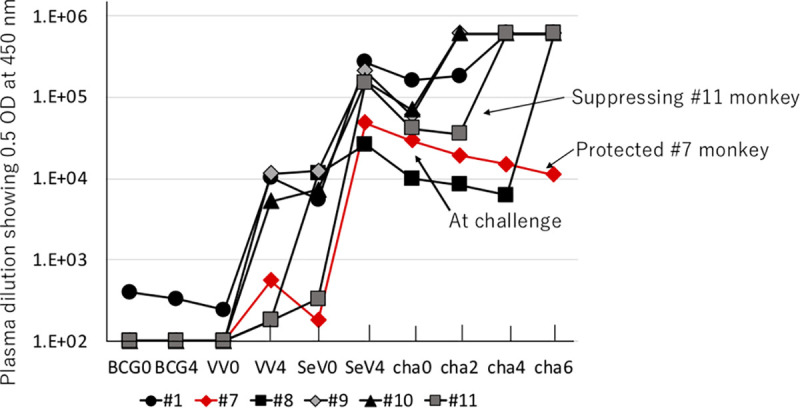
Quantification of anti-Env (gp140) antibodies elicited in the six vaccinated monkeys (monkeys 1, 7, 8, 9, 10, and 11) during the vaccination phase (BCG, VV, and SeV) and after challenge (cha) with SIVmac251. Numbers in the figure show weeks after vaccination or the first challenge. Anti-gp140 antibody titers in the plasma were assessed by ELISA.

To assess the antibodies elicited by the vaccination, anti-SIV Env antibody levels were first determined by titration on gp140-coated enzyme-linked immunosorbent assay (ELISA) plates (Fig. 4). All vaccinees exhibited very similar profiles with respect to induction of anti-Env antibodies during the vaccination phase. rBCG priming did not elicit significant anti-Env antibody. Boosting with rVV elicited a significant level of antibody, and a second SeV boost further elevated the titer 10- to 100-fold more. After the peak (4 weeks postboost), the antibody titer gradually declined but was maintained above 10,000 U/ml until challenge with SIV. After challenge with SIVmac251, monkey 7 did not show any change in anti-Env antibody titer, while in the others, there was a sharp increase. The increased titer in the infected monkeys probably correlated with the acquisition of SIV infection. Importantly, the titers of anti-Env antibody at the first challenge were not correlated with the number of challenges until virus acquisition, exemplified by the fact that the titer of anti-Env antibody in monkey 7 was the fifth highest among six monkeys.

To identify the immunity that correlates with virus acquisition, we further characterized the anti-Env antibodies at the first challenge, including their avidity, neutralizing activity, ability to engender ADCC, and antibody-dependent cellular phagocytosis (ADCP) (Table 1). The avidity of the anti-Env antibody was expressed as the ratio of the amount of anti-Env antibody that retained its binding to gp140 after washing with 9 M urea to the amount retained after washing with phosphate-buffered saline (PBS). While the avidity index did not vary much among the samples, the value for monkey 7 was not higher than that for the other monkeys. Anti-SIV neutralizing antibody activity was evaluated in a TZM-bl cell-based assay. While a significant neutralizing titer against tier 2 SIVmac239 was not detected in 100-fold-diluted plasma from any of the monkeys, activity against tier 1 SIVmac316 was detected to various degrees in the plasma of all the monkeys. However, neutralizing activity was not correlated with protection. ADCC activity was measured using the CEM.NKR CCR5 luc cell target and human NK cell line KHYG-1 effector system (39). The luciferase activity in CEM.NKR CCR5 luc cells, a surrogate marker of infection, was reduced only when both the positive-control anti-SIV Env monoclonal antibody (MAb) B404 (40) and KHYG-1 cells were present, indicating that the system was appropriate for assessing ADCC (data not shown). Significant ADCC activity was not detected in 33-fold-diluted plasma from any of the macaques, although a slight reduction in luciferase activity was noticed. ADCP activity was assayed using fluorescent beads (41), but we did not detect any significant activity with plasma from any of the monkeys. Overall, we did not find any correlation between the anti-Env antibody parameters and protection.

**TABLE 1:** Characteristics of anti-Env antibodies at the first challenge^*a*^

Monkey	Outcome of SIV infection	No. of challenges for SIV acquisition	Anti-Env Ab at first challenge					
			Amt	Avidity index	NT Ab mac239	NT Ab mac316	ADCC	ADCP
1	Infected	2	1.6 × 10^5^	0.45	<100	11,000	<33	<100
7	Sterile protection	>5	2.9 × 10^4^	0.46	<100	1,200	<33	<100
8	Infected	5	1.0 × 10^4^	0.37	<100	720	<33	<100
9	Infected	1	6.0 × 10^4^	0.38	<100	10,100	<33	<100
10	Infected	1	7.0 × 10^4^	0.48	<100	3,900	<33	<100
11	Acquired but suppressed	2	4.1 × 10^4^	0.44	<100	1,010	<33	<100

a The various properties of anti-Env antibodies in the plasma samples prepared at first challenge are characterized as described in Materials and Methods. NT, ●●●.

### SIV-specific T-cell responses induced by vaccination.

**FIG 5:**
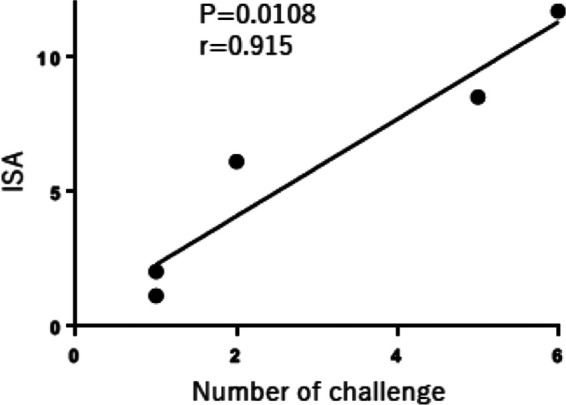
Association between *in vitro* suppression activity (ISA) at first challenge and rate of infection acquisition in immunized animals. Using the two-tailed Spearman rank test, the rate of infection acquisition was found to significantly correlate with the ISA score at first challenge. The graph is plotted as if the protected monkey was infected at the sixth challenge.

The induction of anti-SIV T-cell responses among peripheral blood mononuclear cells (PBMC) over the course of immunization was evaluated in five vaccinated monkeys (PBMC of monkey 1 was not available) by assaying the ability of CD8^+^ T cells to suppress propagation of SIVmac239 in cell culture (*in vitro* suppression activity [ISA]), which is taken to be a direct correlate of *in vivo* suppression of SIV/HIV-1 (42, 43). ISA was evident in monkey 8 after rVV boost and in monkeys 7 and 11 after the second boost with SeV. Then, ISA was maintained until the first challenge with SIV. Monkey 7 had the highest ISA score at the time of challenge, while monkey 8, which had the next highest, required five challenges for virus acquisition. Monkey 11, which had the third highest ISA score, acquired a small amount of SIV after two challenges but had a suppressed viral load below the detection limit (Table 2). In contrast, monkeys 9 and 10, without significant ISA, were infected by the first challenge. Using the two-tailed Spearman rank test, the rate of infection acquisition was found to be significantly correlated with ISA (Fig. 5).

**TABLE 2:** Suppression activities (ISA) of CD8 T cells of vaccinated monkeys during time course^*a*^

Monkey	Outcome of SIV infection	No. of challenges for SIV acquisition	ISA		
			4 wk post-rVV	4 wk post- rSeV	At challenge
1	Infected	2			
7	Sterile protection	>5	2.5	>6.7	11.7
8	Infected	5	8.0	10.8	8.5
9	Infected	1	0.2	1.8	2.0
10	Infected	1	2.2	3.0	1.1
11	Acquired but suppressed	2	3.2	37	6.1

a PBMC were prepared at various points during vaccination, and *in vitro* suppression activity (ISA) was measured as described in Materials and Methods.

**FIG 6:**
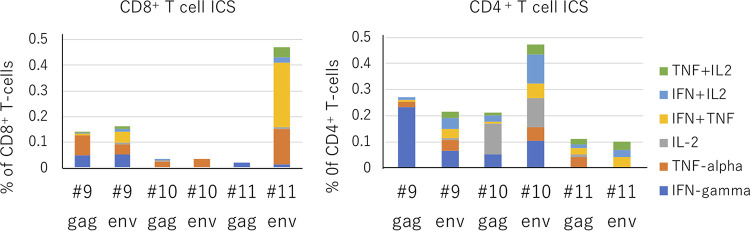
Intracellular staining (ICS) for cytokines in CD4 and CD8 T cells among PBMC from selected monkeys (monkeys 9, 10, and 11) prepared at 4 weeks after boosting with SeV. PBMC were incubated with SIV Gag or SIV Env peptides.

Next, we assessed SIV-specific T cells using an intracellular staining assay (ICS) at 4 weeks after the SeV boost (Fig. 6). Monkey 11 retained more Env-specific multifunctional CD8^+^ T cells, which secreted IFN-γ, tumor necrosis factor alpha (TNF-α), and interleukin 2 (IL-2), than monkeys 9 and 10. In contrast, monkey 11 had fewer of both Env- and Gag-specific CD4^+^ T cells. These results suggest that the immune response elicited by the vaccination made by monkey 11 was skewed to CD8^+^ T-cell induction, leading to suppression of SIV propagation, which is consistent with the high ISA score seen in this monkey after the boost with Env-expressing SeV. Unfortunately, we could not perform ICS in monkeys 1, 7, and 8 because of a paucity of PBMC.

## DISCUSSION

Toward developing a dual vaccine for both tuberculosis and HIV-1, we first compared the pathogenicities of three representative BCG strains, Danish, Pasteur, and Tokyo172, in SCID mice and found the Tokyo172 strain to be the most attenuated. This result is consistent with human Tokyo172 vaccinees experiencing fewer complications than those given other strains of BCG (16). The immunogenicity of Tokyo172 was enhanced by using a urease-deficient mutant, BCGΔurease, in terms of induction of both an anti-BCG response and SIV-specific CD8^+^ T cells, while its pathogenicity was lowered. The increased immunogenicity may be due to easier accessibility of BCG-derived antigens to the route of presentation for CTL induction.

The ability of BCGΔurease to act as a vector for a SIV vaccine was then tested by priming primates with rBCG-SIV, followed by an rVV m8Δ-SIV boost and an SeV-Env second boost. This vaccination had a beneficial effect, resulting in protection of cynomolgus monkeys of Asian origin against SIVmac251 infection. Two animals out of six vaccinees were protected, whereas all seven control animals were infected without any early viral controls. Complete protection of one animal and incomplete but significant suppressive effects on the virus in two animals were observed. That it was indeed complete protection, meaning effective containment before dissemination of systemic infection, was supported by the lack of measurable viremia during the entire follow-up period, there being no increase in anti-Env antibody titer after challenge, and by the lack of detectable viral genomes in any tissues examined.

Immune correlates of protection included the anti-SIV CD8^+^ Tem cells, which possessed suppressive activity, persisted over time, and were present on the day of challenge. Protected animal 7 had the highest ISA score, and the ISA score was significantly correlated with the number of challenges required for SIV acquisition. This association is further supported by our unpublished observation of complete protection from SIVmac251 in an Indian rhesus macaque with an ISA score of >10; furthermore, our results are consistent with reports indicating *in vivo* suppression of SIV/HIV-1 correlates with *in vitro* SIV/HIV-1 suppressive activity, which is probably achieved by killing infected cells (42, 43). We have not fully characterized the target antigens of CD8^+^ T cells in the present study, but the finding in one animal that ISA was detected after the rVV boost, and in two animals that ISA was enhanced by the SeV-Env boost, suggests that Env protein, in addition to Gag protein, is important for inducing effective CTLs. Env-specific CTLs have been recently reported to suppress viral load in SHIV-infected macaques (44).

Anti-Env antibody may not play a major role in protection, because neither the amount, nor the avidity or neutralizing activity, nor the activity in ADCC or ADCP correlated with protection, but it is still conceivable that anti-Env antibodies help SIV-specific CD8^+^ T cells.

To develop a vaccine for HIV, long-lasting immunogenicity, particularly for the maintenance of Tem cells and neutralizing antibodies, is of crucial importance for protection against HIV infection. To elicit and maintain long-lasting anti-HIV/SIV CTLs, including Tem cells, vectors that persist and thus continuously stimulate the immune system would be of particular benefit (10–12). BCG may be a promising persistent vector because of its long track record of safety in humans. The findings herein, and those of our previous study, in which a regimen comprising an rBCG-SIV-Gag prime followed by booster vaccination with m8Δ-SIV-Gag was shown to induce long-lasting Gag-specific IFN-γ^+^, CD107a^+^, and CD8^+^ T cells with the Tem phenotype in mice (25), reinforce the need for further studies on BCG/VV/SeV vaccination as a promising approach for developing an effective HIV-1 vaccine.

## MATERIALS AND METHODS

### Cells and viruses.

Rabbit kidney epithelial cell line RK13, hamster kidney fibroblast line BHK, and human embryonic kidney cell line 293T were cultured in RPMI 1640 medium and Dulbecco’s modified Eagle medium supplemented with 10% fetal bovine serum (FBS). LC16m8Δ and m8Δ-SIV-Gag (m8Δ/pSFJ/SIVGag) viral vectors have been described previously (24, 25). SIVmac239 and SHIV MK1 (45) were produced by transfection of a plasmid harboring each infectious clone into 293T cells and titrated by a TZM-bl cell-based assay (46).

### Construction of vaccinia virus LC16m8Δ expressing SIV genes.

To construct LC16m8Δ expressing the SIV reverse transcriptase/integrase (*pol*) gene (m8Δ-SIV-RT/IN) or SIV *env* gene (m8Δ-SIV-Env), *pol* (nucleotides [nt] 3107 to 5970) and *env* (nt 6860 to 9506) sequences of SIVmac239 (GenBank accession no. AY588945) were amplified by PCR and ligated with the genomic DNA of m8ΔVNC110, followed by transfection into canarypox virus-infected BHK cells, from which the recombinant viruses were recovered (24). To construct m8Δ-SIV-RTN, a fusion gene of *rev*, *tat*, and *nef* was used as a template for PCR (47). Expression of SIV proteins was ascertained by Western blotting using sera from a SIVmac239-infected macaque to detect Gag, RT/IN, and Env proteins and an anti-Nef MAb to detect RTN.

### Construction of a BCGΔurease and expression of SIVmac Gag, Env gp120, or RTN proteins.

The construction of rBCG-SIV-Gag-opt (codon optimized) has been described previously (25). To construct rBCG-SIV-gp120, DNA fragments encoding SIVmac239 gp120 lacking the signal sequence (nt 6929 to 8326) were amplified by PCR using the primers gp120S-F, 5′-CTTTCAATTGCCCTATATGTCACAGTCTTTTATGGTG-3′ (MunI site underlined), and gp120S-R, 5′-CTTTGGGCCCTTATCTTTTTATTTCTTGAGGTGCCAC-3′ (ApaI site underlined), and cloned into a site downstream of the *hsp60* promoter (48) of pUC-hspK (49). To construct rBCG-SIV-RTN, the sequence of the RTN fusion gene encoding amino acid (aa) 78 of Rev to the C terminus of Nef was isolated from pTK-RTN by cutting with PstI and AvaII. A MunI-PstI fragment encoding aa 1 to 77 of Rev was chemically synthesized with codons preferred in BCG and ligated with the PstI-AvaII fragment to clone into pUC-hspK, in which the ApaI site was converted to AvaII by an adaptor. The resultant expression cassette for Env gp120 or RTN was subcloned into pSO246 (50) and used to transform BCGΔurease. The expression of SIV genes was ascertained by Western blotting.

### Construction of Sendai virus expressing SIVmac Env gp160 gene.

Potential EIS (transcription restart sequence) sequences that may affect transcription of SeV were identified in the SIV *env* gene, and nucleotide substitutions that did not alter the amino acid sequence were made using *in vitro* mutagenesis. The mutated *env* fragment was subcloned into pSeV/DF, and replication-incompetent SeVJRCSFenv recombinant virus was constructed as previously described (51). Expression of the *env* gene was ascertained by Western blotting.

### BCG infection of SCID mice.

All mouse experiments were performed in accordance with the guidelines for animal use and experimentation as set out by the National Institutes of Biomedical Innovation, Health, and Nutrition, Japan. The Tokyo172, Danish, or Pasteur strain of BCG (10^7^ CFU/mouse) was inoculated into the tail vein of 5-week-old SCID mice (Charles River Co., Ltd. Tokyo, Japan). Each BCG strain was grown in Middlebrook 7H9 broth containing albumin dextrose catalase (ADC) supplement (Becton, Dickinson and Co., Tokyo, Japan), and a log-phase culture was used for preparing the inoculation. Body weights were measured every several days. To assess infection rates, the lungs and spleens of three mice/group were isolated 0, 3, 6, or 8 weeks after inoculation and homogenized in PBS using a gentleMACS dissociator (Miltenyi Biotec, Tokyo, Japan), and then the number of live bacteria in the spleen samples was measured by colony formation on agar plates. Eight weeks after inoculation, the spleens and lungs from the remaining mice were fixed in 3.6% formalin solution, paraffin embedded, and sectioned for staining with hematoxylin and eosin.

### Immunogenicity of BCGΔurease in mice.

To compare the immunogenicities of wild-type Tokyo172 with Tokyo172Δurease, each harboring plasmid bearing the SIV *gag* gene, five C57BL/6J mice were primed by inoculation with 0.5 mg of either strain. Five weeks later, the mice were boosted with 1 × 10^7^ PFU m8Δ-SIV-Gag by scarification, and then 4 weeks later, splenocytes were prepared. CD4^+^ T and CD8^+^ T cells were isolated with anti-CD4 MAb- or anti-CD8 MAb-coated microbeads (Miltenyi Biotec). For the ELISPOT assay, CD4^+^ and CD8^+^ T cells were suspended at a concentration of 1 × 10^6^ cells/well in culture medium and stimulated with 2 µg/ml peptide 25 (CD4^+^ T-cell epitope of Ag85B, a major protein of BCG, FQDAYNAAGGHNAVF) or GM10 peptide (CD8^+^ T-cell epitope of mycobacterial Mtb32a protein, GAPINSATAM), DD13 peptide (CD4^+^ T-cell epitope of SIV Gag, DRFYKSLRAEQTD), or AL11 peptide (CD8^+^ T-cell epitope of SIV Gag, AAVKNWMTQTL) on ELISPOT plates (Mabtech, Nacka Strand, Sweden). After incubation at 37°C for 24 h, the plates were washed thoroughly with PBS and incubated for 2 h at room temperature with a solution of 1 µg/ml biotinylated anti-mouse IFN-γ MAb R4-6A2 (Mabtech) in PBS containing 0.5% FBS. After the plates had been washed with PBS, 100 µl streptavidin-alkaline phosphatase (Mabtech) at a 1/500 dilution was added to each well. Spots were visualized and counted using a KS ELISPOT reader (Zeiss, Oberkochen, Germany).

### Monkeys, immunization, and challenge.

Cynomolgus monkeys (Macaca fascicularis) and Indian rhesus macaques (Macaca mulatta) were housed and maintained according to the Regulation on Animal Experimentation Guidelines at the National Institutes of Biomedical Innovation, Health and Nutrition and Kyoto University. All experiments with monkeys were approved by the Committee for Experimental Use of Nonhuman Primates at the Tsukuba Primate Research Center and the Institute for Virus Research, Kyoto University. All monkeys were negative for prior exposure to SIV, simian retrovirus type D, and simian T-cell leukemia virus. Their MHCs were determined as described previously (52) and are shown in Tables 3 and 4. Cynomolgus monkeys were immunized subcutaneously at week 0 with rBCGΔurease-SIV-Gag, rBCGΔurease-SIV-gp120, and rBCGΔurease-SIV-RTN (0.5 mg or 2.5 mg/recombinant), boosted 8 weeks later with m8Δ-SIV-Gag, m8Δ-SIV-gp160, m8Δ-SIV-RT/IN, and m8Δ-SIV-RTN (10^7^ PFU/recombinant) by scarification, and then intranasally boosted a second time at 16 weeks with 10^9^ CFU SeV-SIV-gp160 (Fig. 1a). Control monkeys did not receive any vectors. All monkeys were challenged intrarectally at week 24 with a low dose (300 TCID_50_) of the highly pathogenic SIVmac251 challenge stock that was originally obtained from Nancy Miller (NIAID, NIH) and then propagated at Kyoto University in PBMC of Indian rhesus macaques. SIV was administered weekly 5 times to cynomolgus monkeys or to rhesus macaques until plasma viral RNA was positively detected by quantitative PCR. Three Indian rhesus macaques that lacked SIV-resistant MHCs (Table 4) (53) were selected and inoculated with the same stock of SIVmac251 as described above.

**TABLE 3:** MHCs of cynomolgus monkeys used in this study

Monkey	Origin(s)	Sex^*b*^	Age (yr)	MHC class I *Mafa-*A	MHC class I *Mafa-*B	Used for vaccine or control
2	Malaysia/Philippines-Malaysia (mixed)	M	3	A1*068:02	B*038:02:01, B*043:01, B*053:01:01, B*057:03, B*086:01:02, B*092:01:01	Control
3	Malaysia/Indonesia (mixed)	M	3	A1*010:01/02, A1*058:01	B*044:01, B*149:02, B*034:05	Control
12	Malaysia	M	3	A1*006:01:02, A1*089:02, A3*13:03:01	B*030:03:02, B*041:01, B*048:03, B*083:02, B*143:01	Control
13	Indonesia	M	3	A1*041:03:01, A2*05:35, A4*14:11:01:01	B*030:03:02, B*045:01, B*098:02, B*153:01, B*154:01	Control
14	Malaysia	M	3	A1*066:02, A1*068:01, A1*089:02, A2*05:36	B*010:01, B*017:01, B*143:01, B*145:02, B*152:02, B*157:01	Control
0036	Philippines	F	4	A1*008:02, A1*089:02	B*116:01, B*137:03, B*143:01, B*157:01	Control
0037	Indonesia	F	6	A1*010:02:01, A1*062:02	B*044:04, B*140:01, B*154:01, B*157:02	Control
1	Indonesia/Malaysia-?^*a*^ (mixed)	M	3	A1*027:03, A1*059:02, A1*06:06:02	B*029:01:01, B*031:01:01, B*044:01/03, B*059:01	Vaccine
7	Malaysia	M	5	A1*023:01, A1*125*01, A5*30-new, A6*01:02	B*002:03, B*074:02	Vaccine
8	Malaysia/Philippines-Malaysia (mixed)	M	4	A1*008:02, A1*010:01/02	B*081:01, B*082:01, B*162:01	Vaccine
9	Indonesia/Philippines (mixed)	M	3	*A1* *008:02, A1 *010:02:01, A3 *13:02	*B* *033:02, B *075:01, B *095:01, B *098:05, B *143:01	Vaccine
10	Philippines	M	3	*A1* *089:05, A1 *089:02	*B* *033:02, B *095:01, B *136:03, B *137:03	Vaccine
11	Philippines	M	2	*A1* *008:03, A1 *052:02, A1 *100:01	*B* *037:02, B *068:02, B *068:03, B *137:03	Vaccine

a For monkey 1, the maternal grandfather was unknown. The maternal grandmother was from Malaysia.

b M, male; F, female.

**TABLE 4:** MHC of rhesus macaques used in this study

Monkey	Origin	Sex^*a*^	Age (yr)	MHC class I *Mamu-*A	MHC class I *Mamu-*B	MHC class I *Mamu-*I	No. of inoculations for acquisition of SIV
MM634	India	F	4	A1*028-new	B*029:02/01:01, B*043:01, B*060:02	I*01:02:01	7
MM637	India	F	2	A1*007:02, A1*049:02/03/05	B*007:02/03, B*069:01, B*072:01:01	I*01:18	1
MM644	India	M	4	A1*045:01, A1*028-new, A2*05:02:02	B*017:01, B*019:01:01, B*029:01:01/02, B*047:01, B*082:02		4

a M, male; F, female.

### Quantitative PCR of SIV copy number.

Blood, collected into EDTA as an anticoagulant, was taken periodically for quantification of plasma viral RNA load. Tissue samples were obtained at the time of euthanasia to quantify proviral DNA. DNA samples were extracted directly from frozen sections of tissue from each monkey with a DNeasy tissue kit (Qiagen, Tokyo, Japan). The viral load in plasma and the proviral DNA load in lymphoid tissues were determined by quantitative reverse transcription (RT)-PCR and nested PCR, respectively, as described previously (54).

### Anti-gp140 antibody ELISA.

SIV-specific antibody responses were determined as previously described (27). Briefly, ELISA plates were coated with 50 ng per well of soluble gp140 trimeric protein. Serially diluted plasma was assayed for each animal. Horseradish peroxidase (HRP)-conjugated anti-monkey IgGs was used for detection with TMB HRP substrate solution (Bio-Rad, Tokyo, Japan). Absorption was determined at 450 nm using an ELISA plate reader (Perkin Elmer, Tokyo, Japan). SIV-specific antibody titer was quantified as the serum dilution at which an optical density (OD) of 0.5 was achieved. The avidity of anti-SIV Env IgG antibodies was determined by washing with urea as described previously (27). Relative avidity was estimated as the ratio of absorbance after washing with urea to that after washing with PBS.

### Evaluation of neutralizing activity.

The SIV neutralizing activity was measured in a TZM-bl cell-based assay using the *env* genes of SIVmac239 and SIVmac316 (55) as previously described (46).

### Evaluation of ADCC.

ADCC activity in monkey plasma was assayed using SIVmac239-infected CEM.NKR CCR5 luc cells and the human NK cell line KHYG-1 cells expressing human CD16 (39). Briefly, CEM.NKR CCR5 luc cells were infected with SIVmac239 at a multiplicity of infection (MOI) of 0.005 or with SHIV MK1 at an MOI of 0.05. Four days later, 60 μl of 1:10 diluted heat-inactivated plasma and 40 μl medium containing 1 × 10^4^ washed and infected CEM-NKR cells and 100 μl of medium containing 1 × 10^5^ to 4 × 10^5^ KHYG-1 cells were added to each well and incubated at 37°C for 8 h in a CO_2_ incubator. Anti-SIV gp160 MAb B404 (40) was used as a positive control. Then, luciferase activity in each well was measured with a luminometer.

### Evaluation of ADCP.

For assays for ADCP activity (41), 10-μm-diameter BB fluorescent polystyrene beads (Polysciences, Warrington, USA) were coated with SIV-gp140 (Advanced BioScience Laboratories, Rockville, USA) according to the manufacturer’s protocol. SIV-gp140-coated beads (9 × 10^5^) were then opsonized with 100 μl experimental or control plasma diluted 1:100 in serum-free RPMI 1640 for 2 h at 37°C in a U-bottom 96-well plate. Then, 2 × 10^4^ THP-1 cells (a human monocytic leukemia cell line) in 100 μl medium was added as effector cells and cultured overnight. The cells were fixed after washing with Accutase detachment solution (Funakoshi, Tokyo, Japan), and fluorescent cells were counted on a FACSverse (BD Biosciences, Tokyo, Japan).

### Assessment of suppression of SIV propagation by CD8^+^ T cells.

Monkey PBMC were isolated regularly throughout the immunization course and stored viably at −80°C until use. The suppression of SIV propagation activity of CD8^+^ T cells was measured by the modified method described previously (42). Briefly, PBMC were separated into CD8^+^ and CD8^−^ cells with MACS nonhuman primate CD8 microbeads (Miltenyi Biotec, Tokyo, Japan). The CD8^−^ cells were stimulated with 5 μg/ml phytohemagglutinin-L in Iscove’s modified Eagle medium (IMEM) containing 20% FBS, 50 μM 2-mercaptoethanol, and 200 U/ml IL-2 for 2 days and then infected with SIVmac239 at a dose of 136 TCID_50_/10^6^ cells. After culture overnight, the infected cells were washed once with medium and then cocultured with positively selected CD8^+^ T cells at 1:1 in IMEM containing 20% FBS, 50 μM 2-mercaptoethanol, and 200 U/ml IL-2. Five days later, the culture medium was harvested, and the amount of SIV p27 present was measured using a SIV p27 antigen ELISA kit (Fisher Scientific, Tokyo, Japan). The SIV suppression activity (ISA) was calculated as the ratio of the amount of p27 in the samples containing SIV-infected CD8^−^ cells cocultured with CD8^+^ T cells prepared before vaccination to the amount of p27 in the samples containing SIV-infected CD8^−^ cells cocultured with CD8^+^ T cells after vaccination.

### Flow cytometric ICS analysis.

The frequencies of SIV-specific CD4^+^ and CD8^+^ T cells were measured by ICS. PBMC isolated at 4 weeks after boosting with SeV were viably frozen for later analysis. Frozen PBMC were viably thawed and incubated with or without a SIV Gag 15mer peptide or Env 15mer peptide pool in the presence of MAbs against CD49d (clone 9F10) as described previously (11). Then, the cells were stained with fluorescence-conjugated MAbs to CD3 (clone sp34-2), CD4 (clone L200), CD8 (clone SK1), CCR7 (clone 3D12), CD28 (clone 28.2), IFN-γ (clone 4S.B3), IL-2 (clone MQ1-17H12), and TNF-α (Mab11). Flow cytometry analyses were performed on a FACSverse, equipped with FlowJo software (BD Biosciences, Tokyo, Japan).

### Data availability.

Accession numbers from the DNA Data Bank of Japan (DDBJ) for newly identified MHC alleles in this study were as follows: LC547433 for Mamu-A1*028-new (MM634 in Table 4) and LC547434 for Mafa-A5*30-new (monkey 7 in Table 3).

## Supplementary Material

Supplemental file 1
